# Postoperative stiffness and rotator cuff tendon healing: a narrative review

**DOI:** 10.1016/j.xrrt.2025.07.012

**Published:** 2025-08-11

**Authors:** Victor Chen, Julia Beretov, George A.C. Murrell

**Affiliations:** Orthopaedic Research Institute, St George Hospital Campus, University of New South Wales, Sydney, Australia

**Keywords:** Rotator cuff repair, Shoulder stiffness, Frozen shoulder, Retear, Clinical outcomes, Pain, Range of motion, Cytokines

## Abstract

Range of motion restriction or shoulder stiffness is common in rotator cuff tear patients. Shoulder stiffness occurs both preoperatively and postoperatively and contributes to patient dissatisfaction. However, recent studies have suggested that shoulder stiffness may be associated with a greater healing response and lower retear rate. Elevated levels of cytokines such as interleukin (IL)-6, IL-1β, and IL-8 in the joint fluid, synovial tissue, and subacromial bursa of patients with shoulder stiffness suggests an increased healing response following tendon injury. Understanding the mechanisms underlying tendon healing and the development of shoulder stiffness may aid in the development of novel therapies to improve outcomes following rotator cuff surgery.

Rotator cuff tears are a common cause of shoulder pain and loss of function. Surgical repair of rotator cuff tears are often complicated by retears and/or failure to heal.^23^ Importantly patients with retears have poorer shoulder function compared to patients with intact repairs.^6^^,^^26^ Furthermore, the probability of successful repair decreases with successive surgery. A study by Shamsudin et al^29^ found patients who underwent revision cuff repairs were twice as likely to retear compared to patients with primary repairs.

A common complication following rotator cuff repair is postoperative shoulder stiffness.^3^^,^^36^ Whilst rotator cuff repair may be successful from a surgical perspective, loss of range of motion and pain postoperatively can be major contributors to patient dissatisfaction. A study by Zhong et al^38^ on the determinants of patient-rated benefit following surgery found greater patient rated benefit in shoulders that were less stiff at 6 months follow-up. However recent studies have suggested that preoperative and/or postoperative stiffness may be associated with a lower risk of rotator cuff repair retear.^18^^,^^19^^,^^33^ We hypothesize that shoulder stiffness in rotator cuff repair patients may be a temporary byproduct of an increased healing response that, in the long run, contributes to better surgical outcomes.

## Methods

PubMed, Medline, and Google Scholar databases updated to February 2025 were searched. A combination of keywords “rotator cuff” OR “supraspinatus” AND “stiffness” OR “cytokines” was used. Reference lists from papers were also used to find relevant studies. The data were extracted by one author, and studies were verified by another senior author.

## Discussion

### Preoperative stiffness

A study by McGrath et al^18^ at our institution explored the relationship between preoperative shoulder stiffness and retear rates following rotator cuff repair. This cohort study compared outcomes in patients undergoing rotator cuff repair and concurrent capsular release for preoperative stiffness (n = 25) to a matched group receiving only rotator cuff repair (n = 170). The group that underwent concurrent capsular release had a 0% retear rate at both 6 months and 2 years postsurgery, in contrast to retear rates of 14% at 6 months and 20% at 2 years in the group with rotator cuff repair only. Kim et al^10^ conducted a similar cohort study comparing patients with rotator cuff repair and concurrent capsular release (n = 39) to those with rotator cuff repair only (n = 320). Their study found that the group with capsular release had a retear rate of 2% compared to 14% in patients undergoing rotator cuff repair only.

### Postoperative stiffness

A cohort study of 1,533 arthroscopic rotator cuff repairs was conducted at our institution by McNamara et al^19^ who assessed patients preoperatively and at 1, 6, 12, and 24 weeks postoperatively. Retear rate was assessed at 24 weeks using ultrasound. The study found that decreased passive shoulder range of motion at 6-week and 12-week follow-up was correlated with a lower retear rate. However, by 24 weeks, no association was observed between range of motion and retear rates, suggesting that early postoperative stiffness is a transient byproduct of the healing process. Patients in the study were assigned to the shoulder stiffness group if their external rotation was ≤ 20° at 6 weeks follow-up. The retear rate was significantly lower in the stiff group at 6 months, being 7% compared to 15% in the nonstiff group.

In another study at our institution, we examined predictors of retear in a cohort of 1,526 primary arthroscopic rotator cuff repairs.^9^ A multivariate logistic regression was used to identify the independent predictors of retear. Tear size, passive external rotation, and passive forward flexion measured at 6 weeks were the strongest predictors of retear at 6 months. Repair integrity was assessed with ultrasound at 6 months, and shoulder stiffness was determined at 6 weeks follow-up. Tear size and shoulder stiffness had an additive effect, where retear rates were 1% in patients with shoulder stiffness (external rotation ≤ 27°) and a tear ≤1 cm^2^, whilst patients without shoulder stiffness (external rotation < 27°) with tears > 6 cm^2^ had a retear rate of 40%.

A study by Takahashi et al^33^ also explored shoulder stiffness and tendon healing. Their study on 155 primary arthroscopic rotator cuff repairs defined shoulder stiffness at 3 months follow-up as passive forward flexion < 120°, external rotation < 30°, or internal rotation below L3. Tendon integrity was confirmed with magnetic resonance imaging at 12 months and retear rate was 5% in the shoulder stiffness group compared to 17% in the nonstiff group. Pain measured using visual analog scale was significantly higher in the stiff group at 12 months. They also found that functional scores were significantly lower at 3, 6, and 12 months follow-up in group with shoulder stiffness. The results of Takahashi et al,^33^ Guo et al,^9^ and McNamara et al^19^ suggest that whilst shoulder stiffness is associated with lower retear risk, shoulder stiffness is also associated with increased pain and restricted shoulder function.

**Figure 1:**
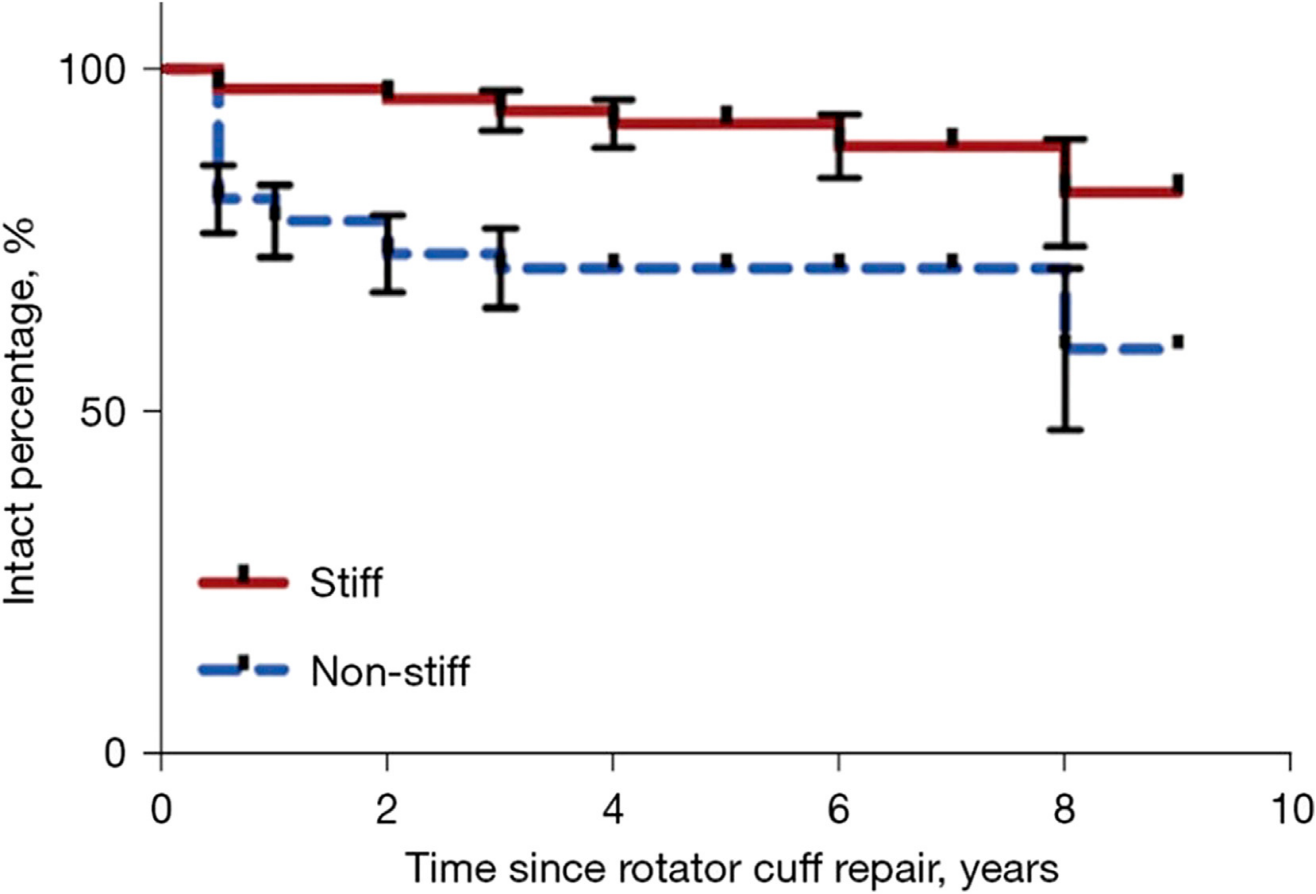
Rotator cuff repair integrity in stiff and nonstiff groups over 9-year follow-up. Figure reproduced with permission from Millican et al.^21^

Millican et al^21^ followed up a portion of patients (n = 132) from the study by McNamara et al^19^ and measured clinical outcomes and repair integrity in patients up to 9 years postsurgery. Stiffness in the study was diagnosed according to external rotation (≤20°) at 6 weeks follow-up. Repairs remained intact to a greater extent in the stiff cohort over the course of 9 years when compared to the nonstiff group (Fig. 1). Although shoulder range of motion was significantly reduced in the stiff group at 6 weeks follow-up, there was no significant difference in internal rotation, abduction, or forward flexion at final follow-up between the stiff and nonstiff groups. Patient-ranked outcomes, including difficulty with overhead activities, difficulty reaching behind the back, and level of shoulder stiffness showed no difference in outcome between the 2 groups. The stiff group did have a reduced external rotation of 51° compared to 60° in the nonstiff group. However, patients with shoulder stiffness were significantly more satisfied with their shoulders at final follow-up when compared to patients in the nonstiff group. The results of Millican et al^21^ suggest that although postoperative stiffness may negatively impact patient satisfaction and clinical outcomes in the short term, these effects are often temporary. Stiffness and pain generally improve over time, with patients in the stiff group ultimately achieving better long-term shoulder function compared to those without stiffness.

### Shoulder stiffness pathogenesis

Histological analysis of capsular tissue from patients with frozen shoulder have consistently demonstrated increased collagen deposition, vascularity, extracellular matrix deposition, neoinnervation, and synovial membrane thickening.^20^ The role of signaling molecules in the pathogenesis of frozen shoulder has been investigated in a preliminary way. A study investigating frozen shoulder by Rodeo et al^27^ stained for cytokines in synovial tissue and found increased expression of interleukin (IL)-lβ and tumor necrosis factor (TNF)- α in patients with frozen shoulder. A study by Akbar et al^1^ cultured capsular fibroblasts from patients with frozen shoulder and found that they produced increased levels of IL-6, IL-8, and chemokine ligand-20. Fibrosis is a fundamental process in the pathogenesis of frozen shoulder and it is thought that fibroblast activation due to cytokine regulation is one mechanism that induces collagen deposition and tissue contracture in the joint capsule.^20^ While studies have investigated the histological features of shoulder stiffness and the cytokines associated with frozen shoulder in capsular tissue, very little research has specifically explored the pathogenesis of shoulder stiffness post–rotator cuff repair and the role that signaling molecules play in tendon healing.

### Rotator cuff tear patients with shoulder stiffness

The involvement of cytokines in rotator cuff tear patients (Box 1) with shoulder stiffness was investigated in a study by Ko et al^13^ The authors compared a group of 14 patients with shoulder stiffness and rotator cuff tears to a control group with rotator cuff tear only. Shoulder stiffness was defined as greater than a 50% loss of passive range of motion, with normal ranges considered to be 180° of forward flexion and abduction and 90° of external and internal rotation. Their study obtained joint fluid and subacromial bursa tissue intraoperatively. Joint fluid from patients with shoulder stiffness had significantly elevated levels of IL-1β (4-fold), IL-6 (1.6-fold), and TNF-α (5-fold) compared to those without shoulder stiffness. In another study, Ko et al^12^ investigated the synovial membrane of the subacromial bursa in patients with shoulder stiffness and rotator cuff tears. Cytokines including IL-8, IL-6, and IL-1β were significantly elevated by approximately 6-fold, 24-fold, and 20-fold, respectively, in the group with shoulder stiffness when compared to patients without stiffness.

A study conducted by Kim et al^11^ investigated the anterior and posterior glenohumeral capsular tissue in rotator cuff repair patients undergoing capsular release for shoulder stiffness, using rotator cuff tear patients without shoulder stiffness as controls. They found significantly higher levels of extracellular matrix and collagen type I and III in the anterior capsules of patients with stiffness compared to the anterior and posterior capsules of control patients. In addition, matrix metalloproteinase (MMP)-2, MMP-9, IL-1, and TGF-β were higher in the anterior capsule when compared to controls.

Substance P, a neuropeptide transmitter, is hypothesized to sensitize postsynaptic neurons to glutamate and facilitate the transmission of pain signals.^39^ The circulating levels of substance P in patients with shoulder stiffness and rotator cuff tear were investigated by Franceschi et al.^5^ Plasma levels of substance P were measured 15 months postsurgery, with patients categorized into stiff and nonstiff groups at that time point. They found that the plasma concentration of substance P was increased by 3.5-fold in the group with shoulder stiffness compared to the control group. It is undetermined if shoulder stiffness at 15 months improves retear rates, where studies by McNamara et al^19^ and Takahashi et al^33^ have shown only early postoperative stiffness to have an impact on retear rates. In addition, serum substance P levels may not accurately reflect intra-articular concentrations.

The association between cytokines and pain was investigated in a study by Okamura et al.^24^ The authors measured the joint fluids of patients undergoing rotator cuff repair for IL-1β, IL-6, and IL-8. They found that IL-8 levels correlated with both IL-6 and IL-1β and that there was a correlation between IL-8 levels and increased preoperative pain scores during rest, movement, and at night. A study by Gotoh et al^7^ found that the level of substance P in the subacromial bursa of rotator cuff repair patients correlated with their preoperative pain levels. Gotoh et al^8^ also found IL-1β levels in the synovium of cuff repair patients correlated with the degree of preoperative pain experienced. Yanagisawa et al^37^ measured vascular endothelial growth factor (VEGF) expression in the subacromial bursa of 38 rotator cuff repair patients and 12 patients with bursitis. VEGF expression was localized to synovial lining cells and vascular endothelial cells. They found that VEGF expression was significantly correlated with preoperative motion pain, synovial proliferation, and mean vessel count and density.

### Cytokines and tendon healing

A study by Tham et al^34^ at our institution investigated the healing process of the rotator cuff tendon in 49 patients, using ultrasound to measure changes at 1 week, 6 weeks, 3 months, and 6 months postoperatively in intact tendons. We observed that posterior capsular thickness was twice that of the unoperated shoulder at 1 week, where it subsequently decreased by 3 and 6 months and eventually showed no difference between operated and nonoperated shoulders at 6 months. We found that whilst the contralateral unoperated tendon exhibited no vascularity, the operated tendon displayed increased vascularity postsurgery, which peaked at 1-week follow-up. Subsequently, tendon vascularity significantly decreased over time, ultimately being nonexistent at 6 month follow-up. The subacromial bursa was another area of interest in the ultrasound study by Tham et al^34^ It was found that subacromial bursa thickness peaked at 1-week postoperatively and subsequently decreased significantly at 6 weeks, 3 months, and 6 months.

Tendons are composed primarily of type I collagen (60%-85% of dry weight) alongside other collagen subtypes, proteoglycans, glycoproteins, and glycosaminoglycans.^25^ The mechanism of retear following surgical repair of the rotator cuff was investigated in a study by Cummins et al.^4^ Their study suggested that the primary mechanism of retear may be the pull-through of an intact suture within the tendon, indicating that improved tendon mechanical strength could play a role in preventing this outcome. A study by Shirachi et al^30^ obtained biopsy samples of torn tendons from 12 patients undergoing rotator cuff repair for full-thickness tears. They measured type I and type III collagen mRNA levels in the samples and evaluated postoperative tendon integrity using magnetic resonance imaging at least 1 year after surgery. The study found that higher levels of type I and type III collagen mRNA were correlated with improved postoperative cuff integrity, suggesting that increased collagen production may enhance tendon tissue quality and contribute to successful healing outcomes.

**Figure 2:**
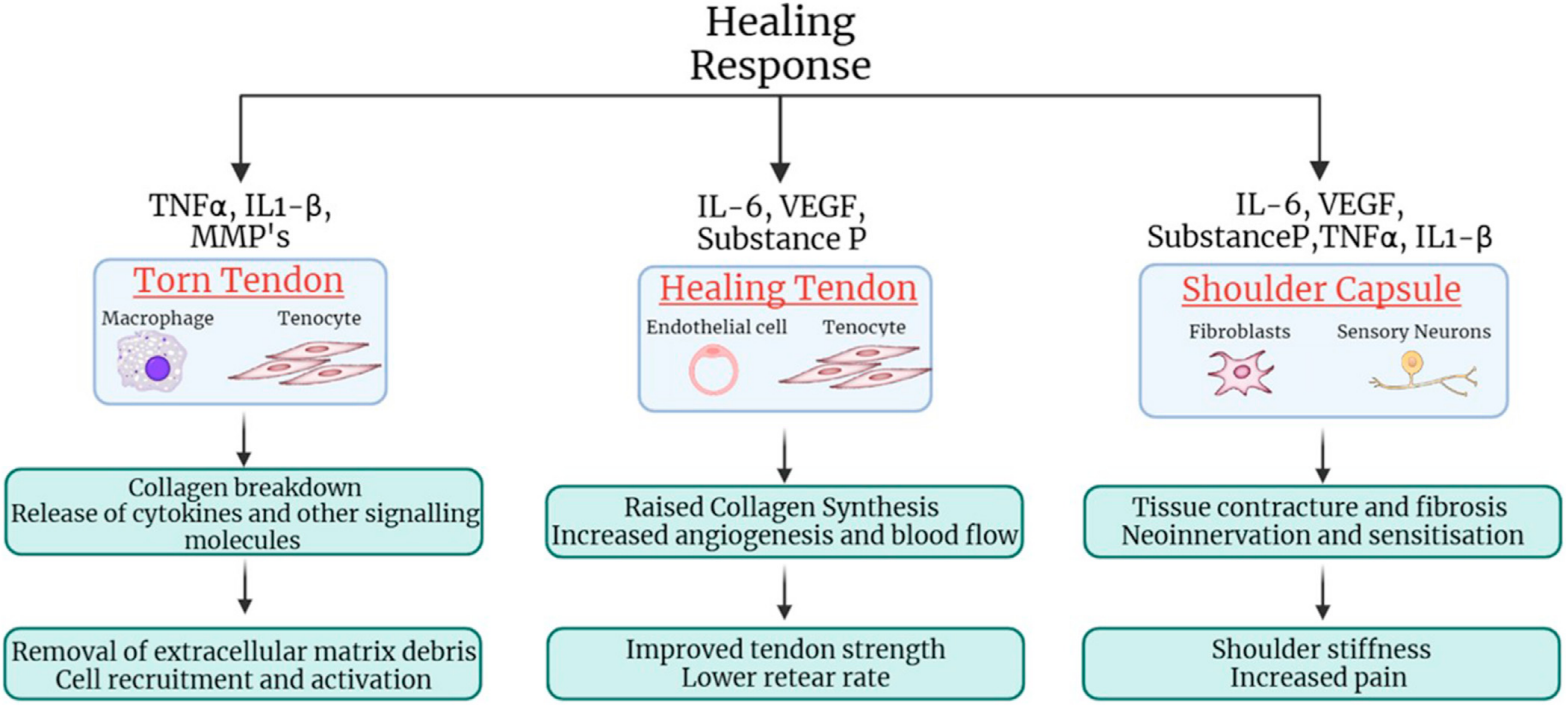
Signaling molecules and rotator cuff healing.

Matthews et al^17^ conducted a histological analysis of 32 torn supraspinatus tendons obtained during mini-open rotator cuff surgery. Their study correlated histological findings with retear rate determined with ultrasound at a mean follow-up time of 35 months postsurgery. Patients who remained intact postrepair demonstrated heightened fibroblast cellularity and proliferation, accompanied by synovial membrane thickening and increased vessel count in the tendons when compared to those that retore. These findings suggest that a robust proliferative response is helpful for successful rotator cuff healing. We hypothesize that signaling molecules in the healing response following repair are upregulated in patients with shoulder stiffness. It is possible that this upregulation of signaling molecules contributes to enhanced repair (Fig. 2).

There have been very few studies exploring the role of cytokines in the healing of human tendons, though there is some evidence in animal and cellular studies. A study by Manning et al^16^ used a canine model to investigate cytokine changes after flexor tendon surgery and measured cytokine level on days 1, 3, and 9 postoperatively. Their study revealed significant early postoperative upregulation of TNF-α, VEGF, IL-1β, and cyclooxygenase-2, which increased 10-fold, 17-fold, 4000-fold, and 800-fold, respectively, in the repaired tendon. Tsuzaki et al^35^ isolated normal human tendon cells from flexor digitorum profundus tendons obtained during surgery and exposed them to 10 pm IL-1β for 16 hours. The study demonstrated that exogenous IL-1β induced the production of endogenous IL-1β, IL-6, cyclooxygenase-2, and MMP-1 and MMP-3. These findings suggest that IL-1β released by inflammatory cells after injury promotes short-term tissue degradation, as MMPs break down collagen and other components of the tendon extracellular matrix. Degradation of collagen by MMPs may facilitate tendon healing by clearing tissue debris and increasing extracellular matrix remodeling.^28^

A study conducted by Lin et al^15^ explored the effect of IL-6 knockout on patellar healing in mice. They found IL-6 knockout mice to have inferior mechanical tendon properties, as measured by decreased maximum stress and normalized modulus when compared to the control group without IL-6 knockout. Stolk et al^31^ investigated the effect of cytokines on tenocytes obtained from ruptured human supraspinatus tendons. The authors stimulated tenocyte and macrophage co-cultures with a mixture of cytokines IL-6, IL-8, monocyte chemoattractant protein-1, TNFα, and interferon-γ—derived from α-cluster of differentiation-3αCD28-stimulated peripheral blood mononuclear cells to mimic inflammation following injury. They noted an elevated release of IL-6 and IL-8 in the co-cultures following stimulation, accompanied with by an increase in collagen I levels. Andersen et al^2^ infused recombinant IL-6 into the peritendinous region of the Achilles tendon in human subjects following a 1-hour treadmill run, using microdialysis to measure procollagen type I NH2-terminal propeptide, a marker of collagen synthesis. The study found that procollagen type I NH2-terminal propeptide concentration was elevated in the IL-6-infused leg, suggesting that IL-6 plays a role in promoting collagen synthesis.

A study by Miura et al^22^ used a rat model to investigate rotator cuff tendon healing with and without the subacromial bursa. They found that following rotator cuff repair, rats with an intact bursa had increased blood vessel formation in the supraspinatus tendon, as well as increased IL-1β, MMP-13, inducible nitric oxide synthase, IL-10, and Arg-1. Notably, collagen III was significantly higher in those with an intact bursa, indicating that cytokines produced by the bursa may improve tendon tissue quality. Sun et al^32^ further investigated the bursa's influence on mechanical tissue quality, finding that rats with an intact bursa had superior maximum load and stiffness following surgery.

IL-4 was investigated alongside IL-6 in the mice study conducted by Lin et al^15^ IL-4 knockout mice had increased normalized modulus and decreased angular deviation, which indicates stiffer tissue with increased collagen organization. These findings suggest that the absence of IL-4 may have some beneficial effects on tendon healing. However, the study noted a significant upregulation of IL-10 and IL13, which may have compensated the lack of IL-4. Both IL-10 and IL-13 are produced by Th2 cells and share similar functional activities.^14^

Box 1 **Signaling molecules elevated in rotator cuff tear patients with shoulder stiffness as compared to patients with rotator cuff tears only.**

Anterior capsular tissue:

• MMP-2 ↑^11^
• MMP-9 ↑^11^
• TNF- α ↓^11^
• TGF-β↑^11^
• Il-1↑^11^

Joint fluid:

• IL-1β ↑^13^
• IL-6 ↑^13^
• TNF- α ↑^13^

Subacromial bursa.

• IL-1β ↑^12^
• IL-6 ↑^12^
• IL-8 ↑^12^

Plasma levels.

• Substance P ↑^5^

## Conclusion

In summary, the mechanisms that are responsible for lower retear rates in patients with shoulder stiffness and rotator cuff tears remain unclear. However, emerging evidence suggests that cytokines and other signaling molecules may play a role in promoting synovial and vascular proliferation and increased glenohumeral capsular thickness, which leads to temporary reductions in shoulder range of motion. The increase in pain and shoulder stiffness following rotator cuff repair may lead to short-term patient dissatisfaction but is associated with improved tendon healing and reduced retear rates at 6 months and beyond.

## Disclaimers

Funding: No funding was disclosed by the authors.

Conflicts of interest: George AC Murrell is a paid consultant with Smith and Nephew (Smith and Nephew products may be used with these studies) and is on the Editorial and/or the governing board of Journal of Shoulder and Elbow Surgery and Shoulder and Elbow (UK). The other authors, their immediate families, and any research foundation with which they are affiliated have not received any financial payments or other benefits from any commercial entity related to the subject of this article.
